# Stage‐Dependent Transcriptional Reprogramming of B‐Cell Receptor Signaling and Antigen Presentation During Bovine Leukemia Virus–Driven Lymphomagenesis

**DOI:** 10.1002/age.70136

**Published:** 2026-06-05

**Authors:** Mohammad Mehdi Akbarin, Zahra Farjami, Cecilia Rodríguez Murillo, Víctor David González‐Fernández, Gabriel Eduardo Acevedo‐Jiménez, Lucero de María Ávila‐De la Vega, Hugo Ramírez Álvarez

**Affiliations:** ^1^ Virology, Genetics, and Molecular Biology Laboratory, Faculty of Higher Studies Cuautitlan, Veterinary Medicine, Campus 4 National Autonomous University of Mexico Cuautitlan Izcalli Mexico; ^2^ Mashhad Medical Sciences‐Medical School Islamic Azad University Mashhad Iran; ^3^ Department of Biology, Damghan Branch Islamic Azad University Damghan Iran

**Keywords:** antigen presentation, B‐cell differentiation, B‐cell receptor signaling, bovine leukemia virus (BLV), lymphoma, RNA sequencing

## Abstract

Bovine leukemia virus (BLV), an oncogenic deltaretrovirus, establishes lifelong infection in cattle and induces enzootic bovine leukosis in a subset of animals following prolonged latency. Despite extensive evidence of immune dysregulation, stage‐specific transcriptional reprogramming of B cells, the primary viral reservoir remains incompletely understood. In this study, we performed a targeted RNA‐sequencing analysis to characterize B‐cell–associated transcriptional profiles across three clinical stages: uninfected controls (CT), asymptomatic BLV‐infected cattle (AC), and persistent lymphocytosis (PL). Differential gene expression analysis revealed a dynamic, stage‐dependent remodeling of B‐cell immune functions. Asymptomatic infection was characterized by significant upregulation of key B‐cell receptor (BCR) signaling components, including *CD79B* and *SYK*, alongside increased expression of major histocompatibility complex class II genes (*BoLA‐DRA, BoLA‐DRB3*) and immunoglobulin assembly gene *JCHAIN*, indicating enhanced antigen responsiveness and partial activation of antibody‐related pathways. However, transcriptional regulators of terminal plasma cell differentiation (*PRDM1* and *XBP1*) remained unchanged, suggesting incomplete maturation. In contrast, progression to persistent lymphocytosis was associated with attenuation of canonical BCR signaling and reduced expression of antigen presentation and immunoglobulin assembly genes relative to the asymptomatic stage. Notably, germinal center–associated transcription factors (*BCL6, AICDA, RGS13, MEF2B*) remained largely unchanged across all comparisons, indicating that BLV‐driven lymphomagenesis does not resemble a classical germinal center–derived process. Collectively, these findings support a biphasic model of BLV‐induced B‐cell modulation, characterized by early immune activation followed by functional reprogramming and immune attenuation during disease progression. This stage‐dependent remodeling provides new mechanistic insights into BLV pathogenesis and B‐cell transformation.

**Abbreviations**
*AICDA*
: Activation‐Induced Cytidine Deaminase
*BANK1*
: B‐Cell Scaffold Protein with Ankyrin Repeats 1
*BCL6*
: B‐Cell Lymphoma 6
*BLK*
: B Lymphocyte Kinase
*BoLA‐DRA*
: Bovine Leukocyte Antigen, Class II, DR Alpha
*BoLA‐DRB3*
: Bovine Leukocyte Antigen, Class II, DR Beta 3
*BTK*
: Bruton Tyrosine Kinase
*CD22*
: Cluster of Differentiation 22
*CD24*
: Cluster of Differentiation 24
*CD27*
: Cluster of Differentiation 27
*CD38*
: Cluster of Differentiation 38
*CD40*
: Cluster of Differentiation 40
*CD79A*
: Cluster of Differentiation 79A (Igα component of B‐cell receptor complex)
*CD79B*
: Cluster of Differentiation 79B (Igβ component of B‐cell receptor complex)
*CD86*
: Cluster of Differentiation 86
*CR2* (CD21): Complement Receptor Type 2
*CXCR5*
: C‐X‐C Motif Chemokine Receptor 5
FPKM: Fragments Per Kilobase of transcript per Million mapped reads
*IGHA1*
: Immunoglobulin Heavy Constant Alpha 1
*IGHD*
: Immunoglobulin Heavy Constant Delta
*IGHG1*
: Immunoglobulin Heavy Constant Gamma 1
*JCHAIN*
: Joining Chain of Multimeric IgA and IgM
*MEF2B*
: Myocyte Enhancer Factor 2B
*MS4A1* (CD20): Membrane Spanning 4‐Domains A1
*MZB1*
: Marginal Zone B and B1 Cell‐Specific Protein
*PAX5*
: Paired Box 5
*PRDM1 (BLIMP1*): PR/SET Domain 1 (B Lymphocyte‐Induced Maturation Protein 1)
*RGS13*
: Regulator of G Protein Signaling 13
*SYK*
: Spleen Tyrosine Kinase
Syndecan 1: *SDC1* (CD138)
*TCL1A*
: T‐Cell Leukemia/Lymphoma 1A
*TNFRSF13B* (*TACI*): Tumor Necrosis Factor Receptor Superfamily Member 13B
TPM: Transcripts Per Million
*XBP1*
: X‐Box Binding Protein 1
**CD19
: Cluster of Differentiation 19**

## 1 Introduction

Bovine leukemia virus (BLV) is an oncogenic deltaretrovirus and the possible etiological agent of enzootic bovine leukosis (EBL), a chronic lymphoproliferative disorder that primarily targets the host immune system (Amato et al. 2023, Cordero‐Pulido et al. 2023). The virus establishes a lifelong persistent infection in cattle by integrating its proviral DNA into the genome of susceptible host cells, predominantly IgM^+^ CD5^+^ B lymphocytes (Castillo‐Rey et al. 2025). While the majority of infected animals remain clinically asymptomatic, approximately 5%–10% eventually progress to malignant lymphosarcoma following a prolonged latency period, highlighting the complex interplay between viral persistence and host immune regulation during disease progression (Gonzalez‐Mendez et al. 2025; Akbarin et al. 2025; Ashrafi et al. 2021).

Accumulating evidence indicates that BLV infection induces profound alterations in host immune homeostasis, particularly within the adaptive immune compartment (Ashrafi et al. 2021) (Gonzalez‐Mendez et al. 2025; Akbarin et al. 2025). Previous transcriptomic analyses have demonstrated that BLV infection is associated with widespread changes in immune‐related gene expression, including modulation of interferon signaling pathways, cytokine networks, and antiviral defense mechanisms (Gonzalez‐Mendez et al. 2025).

More recently, whole‐transcriptome studies have revealed that BLV proviral load is a key determinant of disease phenotype and immunological outcome (Dong et al. 2024; Gonzalez‐Mendez et al. 2025). Animals harboring high proviral loads exhibit extensive transcriptional remodeling, characterized by the differential regulation of genes involved in immune response pathways, cell cycle control, and DNA replication processes (Gonzalez‐Mendez et al. 2025; Akbarin et al. 2025; Dong et al. 2024). Notably, the downregulation of immune‐associated genes in these animals suggests the emergence of an immunosuppressive microenvironment that may facilitate viral persistence and oncogenic transformation.

In our previous RNA‐sequencing study, we demonstrated that BLV infection is accompanied by a progressive shift in host immune responses from cellular to humoral immunity during the lymphocytosis stage (Gonzalez‐Mendez et al. 2025). This transition was marked by the upregulation of MHC class II molecules and anti‐inflammatory cytokines such as IL‐4, IL‐10, and TGF‐β, alongside the downregulation of pro‐inflammatory mediators and pathogen recognition receptors, including TLR3, TLR7, and TLR9 (Gonzalez‐Mendez et al. 2025). Importantly, these findings suggested that BLV preferentially promotes B‐cell activation over T‐cell–mediated immunity, thereby expanding the population of infected target cells and enhancing viral survival within the host (Gonzalez‐Mendez et al. 2025).

Despite these advances, the transcriptional landscape underlying B‐cell differentiation and functional polarization across distinct clinical stages of BLV infection remains poorly understood. Given that B lymphocytes represent both the primary reservoir for viral replication and the cellular origin of leukemic transformation (Gonzalez‐Mendez et al. 2025; Akbarin et al. 2025; Ashrafi et al. 2021), a detailed characterization of B‐cell–specific transcriptional programs is essential for elucidating the mechanisms governing viral latency, immune evasion, and disease progression.

Therefore, the present study aims to extend our previous transcriptomic investigations by focusing specifically on the B‐cell immune profile associated with BLV infection using high‐throughput RNA‐sequencing analysis. By comparing gene expression signatures related to B‐cell lineage commitment, activation, germinal center responses, and plasma cell differentiation across different clinical phenotypes of BLV‐infected cattle, this study seeks to provide novel insights into the molecular pathways that shape host–virus interactions and contribute to lymphoproliferative disease development.

## 2 Materials and Methods

### 2.1 Study Design and Ethical Approval

This study represents a targeted secondary transcriptomic analysis derived from our previously published whole‐transcriptome RNA‐sequencing dataset investigating host immune responses to bovine leukemia virus (BLV) infection (Gonzalez‐Mendez et al. 2025). The original RNA‐seq dataset was generated from peripheral blood leukocytes collected from BLV‐infected and uninfected cattle and analyzed using the *Bos taurus* reference genome (Ensembl ARS‐UCD1.2 assembly).

The present study specifically focuses on B‐cell–associated transcriptional signatures across distinct clinical stages of BLV infection through targeted re‐analysis of previously generated bovine RNA‐seq data. No new RNA‐seq sequencing, genome alignment, or primary transcriptome assembly was performed in the current study.

All experimental procedures, animal handling, RNA extraction, sequencing protocols, and bioinformatic preprocessing were conducted as previously described in the original publication and complied with institutional and national guidelines for animal care. Ethical approval for animal sampling was granted by the Institutional Review Board of the Internal Committee on Animal Use and Experimentation (CICUAE‐FESC) of the National Autonomous University of Mexico (code CICUAE‐FESC. NC 23_35/05–2023).

### 2.2 Animal Selection and Sample Collection

From a screened population of 130 Holstein dairy cows, blood samples were obtained from 18 mid‐lactation cows (third or fourth lactation) from a single dairy farm in Querétaro, Mexico. All animals were under routine veterinary supervision and exhibited no concurrent infectious or metabolic diseases at the time of sampling.

All cows had been vaccinated against the bovine respiratory disease complex (BRDC), including bovine respiratory syncytial virus (BRSV), bovine parainfluenza virus type 3 (BPIV‐3), and bovine herpesvirus 1 (BHV‐1).

BLV serostatus was initially determined by enzyme‐linked immunosorbent assay (ELISA), followed by confirmatory polymerase chain reaction (PCR) and quantitative PCR (qPCR) for proviral load (PVL) determination.

Based on serological, molecular, and hematological assessments, animals were classified into three groups: BLV seronegative controls (*n* = 5), BLV‐infected asymptomatic animals (normal lymphocyte count) (*n* = 7), and BLV‐infected animals with persistent lymphocytosis (high lymphocyte count and clinical signs including weight loss) (*n* = 6).

BLV‐positive animals were classified according to both hematological and molecular criteria. Animals exhibiting persistent lymphocyte counts exceeding 10 000 lymphocytes/μL for at least three consecutive months were categorized as persistent lymphocytosis (PL), based on established BLV diagnostic criteria reported in previous studies. BLV‐infected animals with lymphocyte counts within the normal physiological range were classified as asymptomatic carriers (AC). In addition, proviral load (PVL) quantification by qPCR was incorporated into the classification strategy, as PL animals exhibited markedly higher PVL values compared with AC animals.

Lymphocyte counts were determined using a Corning automated cell counter (Corning, NY, USA), and animals were categorized according to previously established criteria for normal and high‐persistence lymphocyte counts.

Peripheral blood was collected via coccygeal venipuncture using Vacutainer tubes (Becton, Dickinson and Company, Franklin Lakes, NJ, USA). Samples were maintained at 4°C immediately after collection and transported to the laboratory for processing.

### 2.3 Peripheral Blood Leukocyte Isolation

Whole blood was collected into heparinized tubes and centrifuged at 350 × *g* for 15 min to separate plasma and peripheral blood leukocytes (PBLs). Given that BLV infection affects multiple leukocyte populations, including B lymphocytes, T lymphocytes, monocytes, and granulocytes, total peripheral blood leukocytes rather than isolated peripheral blood mononuclear cells (PBMCs) were used for downstream analysis.

Peripheral blood leukocytes (PBLs) were isolated prior to RNA extraction to enrich immune‐cell–associated transcripts and reduce erythrocyte‐derived background RNA, thereby improving the sensitivity of leukocyte‐specific transcriptomic profiling. This approach was considered particularly important for evaluating B‐cell–associated immune pathways during BLV infection.

Erythrocytes were removed using a lysis solution as previously described. Plasma and isolated PBLs were stored at −70°C until RNA extraction.

### 2.4 RNA Extraction and Quality Assessment

Approximately 5 × 10^6^ leukocytes per sample were resuspended in 350 μL of RLT buffer supplemented with RNAlater (Thermo Fisher Scientific, Waltham, MA, USA; Cat. No. AM7020) to ensure effective cell lysis and RNA stabilization.

Total RNA was extracted using the FavorPrep Total RNA Isolation Kit II (FAVORGEN, Ping‐Tung, Taiwan) following the manufacturer's protocol. RNA concentration and purity were assessed using a NanoDrop One C microvolume UV–Vis spectrophotometer (Thermo Fisher Scientific, Waltham, MA, USA). All samples met quality standards for downstream sequencing. Purified RNA was stored at −80°C in RNase‐free water until library preparation.

### 2.5 BLV Serology and Proviral Load Quantification

BLV seropositivity was determined using a commercial ELISA kit (VMRD, Pullman, WA, USA). Samples testing positive for BLV antibodies were subsequently analyzed by quantitative PCR to determine the infection.

Based on BLV seropositivity, PVL, and lymphocyte count, BLV‐positive animals were further categorized into asymptomatic and persistent lymphocytosis phenotypes (more than 50% for 3 months).

### 2.6 Library Preparation and RNA Sequencing

All 18 samples were selected for RNA sequencing based on serological status, PVL quantification, and clinical classification.

Library preparation was performed by a commercial sequencing provider. Messenger RNA (mRNA) was isolated from total RNA using poly‐T oligo–attached magnetic beads. Purified mRNA was fragmented, and complementary DNA (cDNA) was synthesized using random hexamer primers and dNTPs. After adapter ligation, size selection, amplification, and purification were performed according to standard Illumina protocols.

Sequencing was conducted on the Illumina NovaSeq 6000 platform (Illumina Inc., San Diego, CA, USA), generating paired‐end reads of 150 base pairs. An average of approximately 29 million read pairs per sample was obtained.

### 2.7 Quality Control and Genome Alignment

Raw sequencing data were generated in FASTQ format. Quality assessment was performed using FastQC, and adapter sequences and low‐quality reads were removed prior to downstream analysis.

Cleaned reads were aligned to the *Bos taurus* reference genome (Ensembl ARS‐UCD1.2 assembly) using the HISAT2 alignment algorithm. Gene‐level read counts were quantified using featureCounts.

To evaluate reproducibility and sample consistency, Pearson correlation analyses were performed within biological groups. All correlation coefficients exceeded 0.92, indicating high experimental reproducibility and sample quality.

### 2.8 Differential Gene Expression Analysis

Differential gene expression analysis was conducted using the DESeq2 R package (version 1.20.0, Bioconductor). Raw count data were normalized internally by DESeq2 using its median‐of‐ratios method.

Genes were considered differentially expressed if they met the following criteria: Adjusted *p*‐value (Benjamini–Hochberg false discovery rate, FDR) ≤ 0.05, Absolute log_2_ fold change ≥ 1.

Comparisons were performed between: Control versus Asymptomatic BLV‐infected cattle, Control versus Persistent lymphocytosis cattle, and Asymptomatic versus Persistent lymphocytosis cattle.

Hierarchical clustering of differentially expressed genes was performed using normalized expression values. For visualization and comparative analyses, expression values were transformed to relative *Z*‐scores.

To reduce background noise, genes were retained for downstream analysis if they exhibited expression levels corresponding to FPKM > 1 in at least one sample or transcript per million (TPM) > 0.5 in at least 50% of samples.

### 2.9 Targeted B‐Cell Immune Gene Panel Analysis

To systematically characterize B‐cell–associated transcriptional programs during BLV infection, a curated panel of genes representing canonical stages of B‐cell development, activation, germinal center differentiation, plasma cell maturation, immunoglobulin production, and antigen presentation was selected based on established immunological literature. The panel included lineage‐defining markers (e.g., *CD19, PAX5*), core B‐cell receptor (BCR) signaling components (*CD79A, CD79B, SYK, BTK*), activation and co‐stimulatory molecules (*CD40, CD86, CD38, CD27*), germinal center regulators (*BCL6, AICDA, RGS13, MEF2B*), plasma cell differentiation factors (*PRDM1, XBP1, SDC1, MZB1*), immunoglobulin isotypes and assembly genes (*IGHG1, IGHA1, IGHD, JCHAIN*), and antigen presentation molecules (*BoLA‐DRA, BoLA‐DRB3*).

The complete list of genes, their functional classification, biological roles, and supporting references is provided in Table 1. This targeted gene set enabled focused evaluation of stage‐specific transcriptional remodeling of B‐cell functional pathways during BLV infection.

**TABLE 1:** B‐cell immune gene panel with functional classification and references.

Gene symbol	Full gene name	Functional category	Biological role	Key references
*CD19*	Cluster of Differentiation 19	B‐cell lineage/BCR signaling	Core B‐cell marker; amplifies BCR signaling	Kurosaki et al. (2010)
*CD79A*	Cluster of Differentiation 79A (Igα)	BCR signaling	Component of BCR complex required for signal transduction	Reth (2013, 2014)
*CD79B*	Cluster of Differentiation 79B (Igβ)	BCR signaling	Component of BCR complex; mediates antigen‐induced signaling	Reth (2013, 2014)
*MS4A1 (CD20)*	Membrane Spanning 4‐Domains A1	B‐cell lineage marker	Surface marker of mature B cells; regulates Ca2+ signaling	Tedder and Engel (1994)
*CD22*	Cluster of Differentiation 22	BCR regulation	Inhibitory co‐receptor modulating BCR activation	Nitschke (2009)
*CD24*	Cluster of Differentiation 24	Transitional B‐cell marker	Marker of immature/transitional B cells	Carroll (2004)
*PAX5*	Paired Box 5	Lineage commitment	Master regulator of B‐cell identity	Nutt et al. (2015)
*BANK1*	B‐Cell Scaffold Protein with Ankyrin Repeats 1	BCR signaling adaptor	Scaffold linking BCR signaling pathways	Yokoyama (2002)
*BLK*	B Lymphocyte Kinase	Src‐family kinase	Initiates BCR proximal signaling	Ichikawa‐Tomikawa et al. (2023)
*IGHD*	Immunoglobulin Heavy Constant Delta	Naïve B‐cell marker	Encodes IgD heavy chain	Dirks et al. (2023)
*TCL1A*	T‐Cell Leukemia/Lymphoma 1A	B‐cell survival/oncogenesis	Associated with lymphoid proliferation and malignancy	Aggarwal et al. (2009)
*CD38*	Cluster of Differentiation 38	Activation marker	Activation and plasma cell precursor marker	Malavasi et al. (2008)
*CXCR5*	C‐X‐C Motif Chemokine Receptor 5	Germinal center homing	Controls migration to germinal centers	Allen et al. (2007)
*CD27*	Cluster of Differentiation 27	Memory B‐cell marker	Defines memory B‐cell subsets	Deenick and Tangye (2007)
*TNFRSF13B (TACI)*	Tumor Necrosis Factor Receptor Superfamily Member 13B	Class switching regulator	Regulates immunoglobulin class switching and plasma survival	Mackay et al. (2012)
*CR2 (CD21)*	Complement Receptor Type 2	BCR co‐receptor	Enhances BCR signaling via complement binding	Carroll (2004)
*SDC1 (CD138)*	Syndecan 1	Plasma cell marker	Surface marker of terminal plasma cells	Fakan et al. (2002)
*XBP1*	X‐Box Binding Protein 1	Plasma cell differentiation	Controls unfolded protein response in antibody‐secreting cells	Iwakoshi et al. (2003)
*PRDM1 (BLIMP1)*	PR/SET Domain 1	Plasma cell master regulator	Master transcription factor of plasma cell differentiation	Shapiro‐Shelef and Calame (2005)
*MZB1*	Marginal Zone B and B1 Cell‐Specific Protein	Antibody secretion	Enhances immunoglobulin folding and secretion	Flach et al. (2010)
*IGHG1*	Immunoglobulin Heavy Constant Gamma 1	Immunoglobulin isotype	Encodes IgG1 heavy chain	Stavnezer et al. (2008)
*IGHA1*	Immunoglobulin Heavy Constant Alpha 1	Immunoglobulin isotype	Encodes IgA heavy chain	Stavnezer et al. (2008)
*JCHAIN*	Joining Chain of Multimeric IgA and IgM	Antibody assembly	Required for polymeric IgM and IgA formation	Brandtzaeg (2013)
*BCL6*	B‐Cell Lymphoma 6	Germinal center regulator	Master regulator of germinal center reaction	Victora and Nussenzweig (2012)
*AICDA*	Activation‐Induced Cytidine Deaminase	Somatic hypermutation	Required for class‐switch recombination and SHM	Muramatsu et al. (2000)
*RGS13*	Regulator of G Protein Signaling 13	GC migration regulator	Modulates germinal center B‐cell positioning	Shih et al. (2002)
*MEF2B*	Myocyte Enhancer Factor 2B	GC‐associated TF	Transcription factor enriched in germinal center B cells	Ying et al. (2013)
*BTK*	Bruton Tyrosine Kinase	BCR signaling kinase	Key kinase downstream of BCR activation	Hendriks et al. (2014)
*SYK*	Spleen Tyrosine Kinase	BCR proximal kinase	Initiates BCR‐mediated signal transduction	Kurosaki et al. (2010)
*CD40*	Cluster of Differentiation 40	T‐cell interaction	Mediates T‐cell–dependent B‐cell activation	Elgueta et al. (2009)
*CD86*	Cluster of Differentiation 86	Costimulation molecule	Provides costimulatory signals to T cells	Sharpe and Freeman (2002)
*BoLA‐DRA*	Bovine MHC Class II DR Alpha	Antigen presentation	Encodes MHC class II alpha chain for antigen presentation	Neefjes et al. (2011)
*BoLA‐DRB3*	Bovine MHC Class II DR Beta 3	Antigen presentation	Encodes polymorphic MHC class II beta chain; linked to BLV susceptibility	Juliarena et al. (2008)

*Note:* Genes not included in this immune‐focused panel were excluded from further interpretation in this targeted analysis.

## 3 Results

### 3.1 BLV Proviral Load Distinguishes Asymptomatic and Persistent Lymphocytosis Groups

Analysis of BLV proviral load demonstrated clear molecular differences between the infected groups. The asymptomatic carrier (AC) group exhibited a mean PVL of 5.09 × 10^5^ ± 2.04 × 10^5^ copies/μL, whereas the persistent lymphocytosis (PL) group showed substantially higher PVL values (2.34 × 10^6^ ± 1.02 × 10^6^ copies/μL). These findings further supported the classification of infected animals into asymptomatic and PL phenotypes in combination with hematological criteria.

### 3.2 Differential B‐Cell Immune Gene Expression Across BLV Infection Stages

To characterize the B‐cell immune transcriptional profile associated with BLV infection, we analyzed the differential expression of lineage, activation, germinal center, and plasma cell–associated genes across three clinical comparisons: control versus asymptomatic, control versus persistent lymphocytosis, and persistent lymphocytosis versus asymptomatic animals (File S1).

### 3.3 PVL Findings in AC and PL Groups

Analysis of PVL across the samples showed that the mean proviral load in the asymptomatic carrier (AC) group was 5.09 × 10^5^ ± 2.04 × 10^5^ copies/μL, whereas the persistent lymphocytosis (PL) group exhibited a higher mean value of 2.34 × 10^6^ ± 1.02 × 10^6^ copies/μL (95% CI). Based on these measurements, the samples were subsequently categorized into low and high PVL groups.

### 3.4 Control Versus Asymptomatic BLV Infection

**FIGURE 1:**
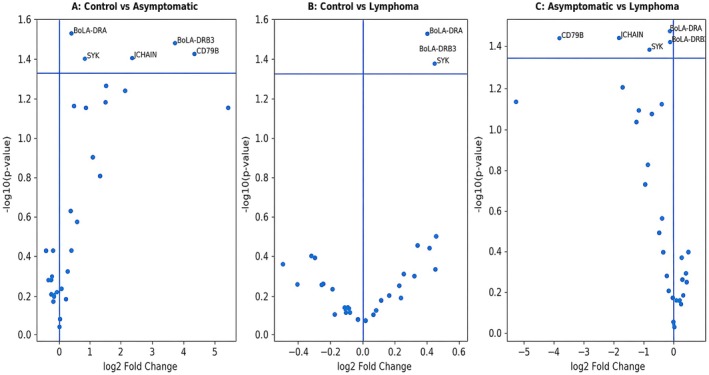
Differential expression of B‐cell–associated immune genes across BLV infection stages. Volcano plots display the log_2_ fold change (log_2_FC) on the *x*‐axis and the –log_10_ (*p*‐value) on the *y*‐axis for selected B‐cell immune profile genes. The horizontal dashed line indicates the statistical significance threshold (*p* = 0.05), and the vertical line represents log_2_FC = 0. Genes with *p* < 0.05 are labeled in each panel. In asymptomatic animals (Panel A), significant upregulation of *CD79B, SYK, JCHAIN, BoLA‐DRA*, and *BoLA‐DRB3* was observed, indicating enhanced B‐cell receptor signaling and antigen presentation activity. In Persistence lymphocytosis animals (Panel B), significant alterations were primarily observed in signaling and antigen presentation pathways. Direct comparison between asymptomatic and Persistence lymphocytosis animals (Panel C) revealed downregulation of B‐cell receptor components and antibody assembly genes in Persistence lymphocytosis cases.

In asymptomatic BLV‐infected cattle, several B‐cell receptor (BCR)–associated genes were upregulated compared with controls. Notably, *CD79B* showed significant upregulation (logFC = 3.69, *p* < 0.05), along with increased expression of SYK (logFC = 1.10, *p* < 0.05), indicating enhanced BCR signaling activity (Figure 1).

Additionally, genes related to antibody secretion and plasma cell differentiation, including *J‐ CHAIN* (logFC = 1.98, *p* < 0.05), were significantly elevated. MHC class II genes *BoLA‐DRA* and *BoLA‐DRB3* were also significantly upregulated (*p* < 0.05), suggesting increased antigen presentation capacity in asymptomatic animals (Figure 1A).

Although not statistically significant, several lineage markers such as *CD19, CD79A, CD22*, and *CD24* exhibited increased expression trends, indicating expansion or activation of B‐cell populations during the asymptomatic stage (Figure 1A).

In contrast, germinal center–associated genes (*BCL6, AICDA, RGS13, MEF2B*) did not demonstrate significant changes.

### 3.5 Control Versus Persistence Lymphocytosis

In Persistence lymphocytosis animals, the transcriptional profile differed from asymptomatic animals. Core B‐cell lineage markers (*CD19, CD79A, CD79B, MS4A1*) did not show significant upregulation relative to controls (Figure 1B).

However, signaling molecules *SYK* (*p* < 0.05) and antigen presentation genes *BoLA‐DRA* (*p* < 0.05) were significantly increased, indicating sustained BCR signaling and immune activation even in malignant stages (Figure 1B).

Plasma cell–associated genes (*PRDM1, XBP1, SDC1, IGHG1, JCHAIN*) did not demonstrate statistically significant differences compared to controls, suggesting that PL development is not characterized by a classical terminal plasma cell differentiation program (Figure 1B).

### 3.6 Persistence Lymphocytosis (PL) Versus Asymptomatic

Direct comparison between PL and asymptomatic animals revealed a marked downregulation of several BCR components in PL cases. *CD79B* (logFC = −3.51, *p* < 0.05) and *SYK* (*p* < 0.05) were significantly reduced in PL compared to asymptomatic cattle, indicating attenuation of BCR signaling during progression to persistent lymphocytosis (Figure 1C).

Similarly, *JCHAIN* expression was significantly lower in PL animals (*p* < 0.05), suggesting reduced immunoglobulin assembly and antibody‐secreting activity compared to the asymptomatic stage (Figure 1C).

MHC class II genes *BoLA‐DRA* and *BoLA‐DRB3* were also significantly decreased in PL compared with asymptomatic animals (*p* < 0.05), implying impaired antigen presentation capacity in advanced disease (Figure 1C).

Most germinal center–related transcription factors (*BCL6, AICDA, RGS13, MEF2B*) remained unchanged between these two infected groups (Figure 1C).

### 3.7 Overall Transcriptional Pattern

Collectively, these findings may indicate that asymptomatic BLV infection is associated with enhanced B‐cell receptor signaling, increased antigen presentation, and partial activation of plasma cell–associated pathways. In contrast, PL development is characterized by relative suppression of BCR signaling components and antibody assembly genes compared to the asymptomatic stage, despite persistent activation of selected immune signaling pathways.

Direct comparison between asymptomatic and persistent lymphocytosis animals revealed attenuation of several B‐cell receptor signaling components and antigen presentation genes during progression to the persistent lymphocytosis stage.

### 3.8 B‐Cell Immune Profiling Reveals Stage‐Dependent Functional Reprogramming During BLV Infection

To better understand how BLV infection reshapes B‐cell functional programs across disease stages, we examined transcriptional signatures associated with B‐cell receptor (BCR) signaling, germinal center activity, plasma cell differentiation, and antigen presentation. Comparative analysis across control, asymptomatic, and PL groups revealed a dynamic and stage‐dependent pattern of B‐cell immune modulation.

#### 3.8.1 Enhanced BCR Signaling Activity in Asymptomatic Infection

In the asymptomatic stage, B cells exhibited clear evidence of enhanced activation. Core components of the BCR signaling complex, including *CD79B* (cluster of differentiation 79B, immunoglobulin beta chain) and *SYK* (spleen tyrosine kinase), were significantly upregulated compared with uninfected controls. These molecules are essential mediators of proximal BCR signaling and antigen‐induced activation, indicating that BLV infection in its clinically silent phase is associated with increased B‐cell signaling competence rather than immune suppression. Supporting this observation, increased expression of major histocompatibility complex class II genes, including *BoLA‐DRA* (major histocompatibility complex, class II, DR alpha) and *BoLA‐DRB3* (major histocompatibility complex, class II, DR beta 3), suggests an augmented antigen presentation capacity during early infection (Table 2).

**TABLE 2:** B‐cell functional profiling summary.

Functional category	Gene symbol	Full gene name	Control versus asymptomatic	Control versus PL	PL versus asymptomatic	Biological interpretation
BCR Signaling	*CD79B*	Cluster of Differentiation 79B (Igβ)	↑ Significant	NS	↓ Significant	Enhanced proximal BCR activation in asymptomatic stage; reduced signaling in lymphoma
BCR Signaling	*SYK*	Spleen Tyrosine Kinase	↑ Significant	NS	↓ Significant	Active antigen‐induced signaling early; attenuated in malignancy
BCR Signaling	*CD79A*	Cluster of Differentiation 79A (Igα)	↑ Trend	NS	NS	Partial activation of BCR complex components
BCR Signaling	*CD19*	Cluster of Differentiation 19	↑ Trend	NS	NS	Amplification of BCR signaling in asymptomatic animals
Immunoglobulin Assembly/Plasma Cell Activity	*JCHAIN*	Joining Chain of Multimeric IgA and IgM	↑ Significant	NS	↓ Significant	Increased antibody assembly early; impaired in PL
Immunoglobulin Assembly/Plasma Cell Activity	*PRDM1*	PR Domain Zinc Finger Protein 1 (BLIMP1)	NS	NS	NS	No evidence of full plasma cell terminal differentiation
Immunoglobulin Assembly/Plasma Cell Activity	*XBP1*	X‐Box Binding Protein 1	NS	NS	NS	Absence of unfolded protein response–driven plasma cell program
Germinal Center Program	*BCL6*	B‐Cell Lymphoma 6 Protein	NS	NS	NS	No strong GC transcriptional signature
Germinal Center Program	*AICDA*	Activation‐Induced Cytidine Deaminase	NS	NS	NS	No significant class‐switch recombination activation
Germinal Center Program	*RGS13*	Regulator of G Protein Signaling 13	NS	NS	NS	GC migration/signaling unchanged
Germinal Center Program	*MEF2B*	Myocyte Enhancer Factor 2B	NS	NS	NS	GC‐associated transcription factor stable
Antigen Presentation (MHC Class II)	*BoLA‐DRA*	Bovine Major Histocompatibility Complex, Class II, DR Alpha	↑ Significant	NS	↓ Significant	Enhanced antigen presentation in asymptomatic stage; reduced in PL
Antigen Presentation (MHC Class II)	*BoLA‐DRB3*	Bovine Major Histocompatibility Complex, Class II, DR Beta 3	↑ Significant	NS	↓ Significant	Suggests immune surveillance early and possible immune evasion later

Abbreviations: ↑, Increase; ↓, Decrease; NS, non‐significant.

#### 3.8.2 Partial Plasma Cell–Associated Activation Without Terminal Differentiation

Evidence of antibody‐related activation was also observed in asymptomatic animals. *JCHAIN* (joining chain of multimeric IgA and IgM) was significantly upregulated, consistent with enhanced immunoglobulin assembly activity. However, key transcriptional regulators of terminal plasma cell differentiation, including *PRDM1* (PR domain zinc finger protein 1; also known as BLIMP1) and *XBP1* (X‐box binding protein 1), were not significantly altered. This indicates that although B cells exhibit functional activation and increased immunoglobulin‐associated machinery, they do not fully transition into a terminal plasma cell phenotype at this stage. Instead, the transcriptional landscape reflects an activated but non‐terminally differentiated B‐cell state (Table 2).

#### 3.8.3 Attenuation of BCR Signaling in Persistent Lymphocytosis

In contrast, progression to PLwas associated with attenuation of physiological B‐cell signaling pathways. Direct comparison between asymptomatic and PL samples demonstrated significant downregulation of *CD79B* and *SYK*, indicating suppression or dysregulation of canonical BCR signaling in PBL. Additionally, *JCHAIN* expression was significantly reduced in PL relative to asymptomatic animals, suggesting impaired immunoglobulin assembly capacity during tumor progression (Table 2). This shift implies that progression to persistent lymphocytosis is accompanied by loss of coordinated immune activation programs that characterize the asymptomatic phase.

#### 3.8.4 Germinal Center Program Remains Largely Unchanged

Notably, classical germinal center–associated transcription factors, including *BCL6* (B‐cell lymphoma 6 protein), *AICDA* (activation‐induced cytidine deaminase), *RGS13* (regulator of G protein signaling 13), and *MEF2B* (myocyte enhancer factor 2B), did not show significant differential expression across comparisons (Table 2). This finding suggests that BLV‐associated lymphomagenesis does not transcriptionally resemble a conventional germinal center–derived lymphoma phenotype. Rather, the disease appears to involve functional reprogramming of activated peripheral B cells without strong engagement of a canonical germinal center program.

#### 3.8.5 Altered Antigen Presentation Dynamics During Disease Progression

Alterations in antigen presentation pathways further highlight stage‐dependent immune remodeling. MHC class II–associated genes, including *BoLA‐DRA* and *BoLA‐DRB3*, were significantly upregulated in asymptomatic animals compared with controls, suggesting enhanced antigen presentation activity during early BLV infection.

This pattern suggests that early infection is characterized by enhanced antigen presentation and immune surveillance, whereas malignant progression may involve partial impairment of antigen‐presenting capacity, potentially contributing to immune evasion (Table 2).

Taken together, these findings define a biphasic model of B‐cell immune modulation during BLV infection. The asymptomatic stage is characterized by heightened BCR signaling, increased antigen presentation, and partial activation of antibody‐related machinery. In contrast, PL development is associated with transcriptional reprogramming that attenuates physiological B‐cell signaling and immune effector functions. This stage‐dependent shift from immune activation to functional dysregulation may represent a critical transition in BLV‐driven oncogenesis.

## 4 Discussion

The present study provides a focused transcriptomic characterization of B‐cell–associated immune programs across distinct stages of BLV infection and demonstrates a stage‐dependent functional reprogramming of B cells during disease progression. Our findings support a biphasic model in which asymptomatic infection is characterized by enhanced B‐cell receptor (BCR) signaling and antigen presentation, whereas PL development is associated with attenuation of canonical BCR signaling and partial impairment of immune effector functions.

### 4.1 Enhanced BCR Signaling During Asymptomatic Infection

**FIGURE 2:**
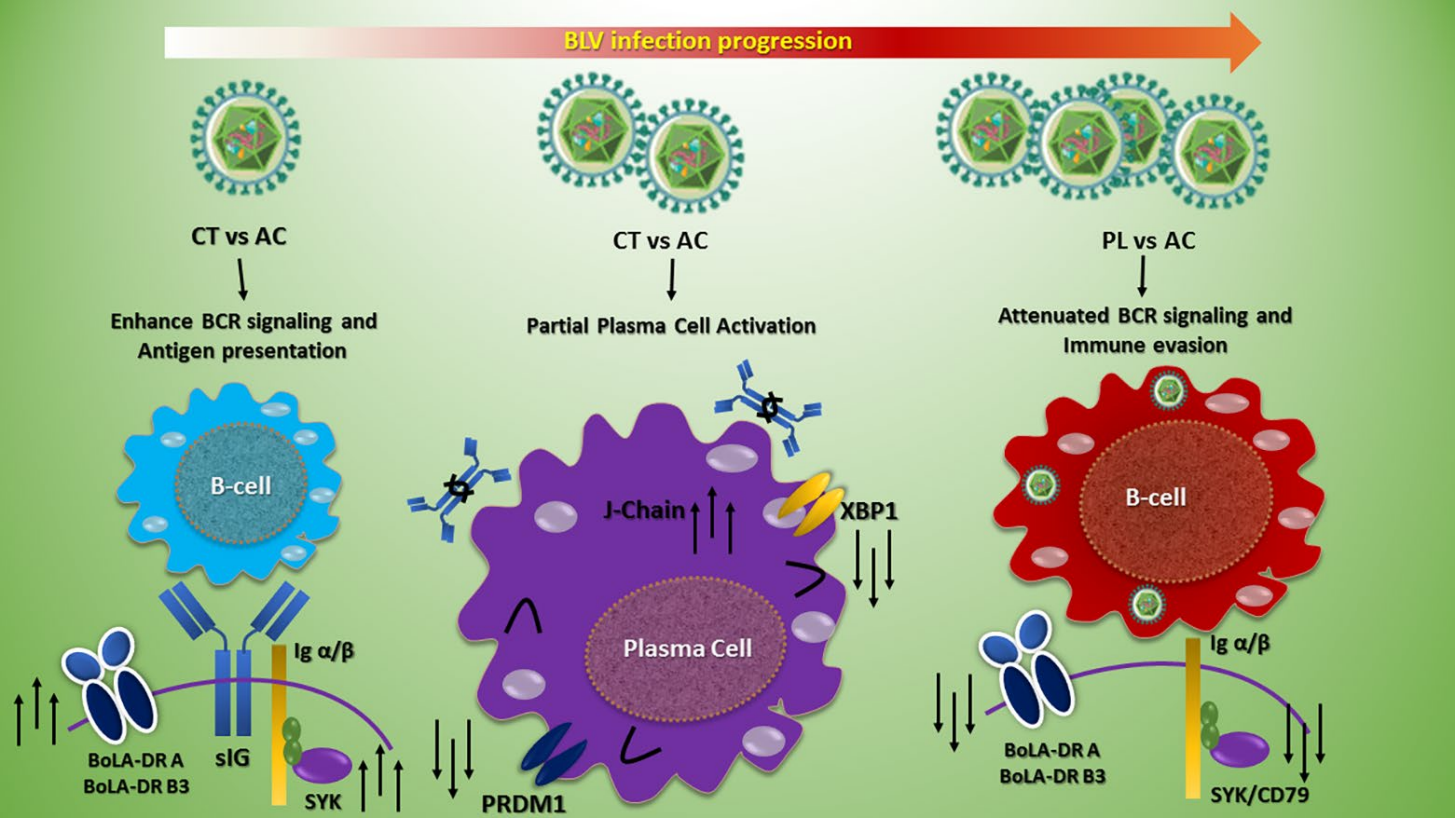
Stage‐dependent remodeling of B‐cell functional programs during bovine leukemia virus (BLV) infection. Schematic representation of transcriptomic changes in B cells across three stages of bovine leukemia virus (BLV) infection: CT versus AC, CT versus PL, and PL versus AC. In the control vs. asymptomatic stage, B cells exhibit enhanced B‐cell receptor (BCR) signaling and increased antigen presentation capacity. This is illustrated by upregulation of *Igα/Igβ* (immunoglobulin alpha and beta chains; *CD79A/CD79B*) and *SYK* (spleen tyrosine kinase), together with increased expression of Bovine MHC class II (major histocompatibility complex class II) molecules, including *BoLA‐DRA* (major histocompatibility complex, class II, DR alpha chain) and *BoLA‐DRB3* (major histocompatibility complex, class II, DR beta 1 chain). These changes indicate heightened antigen responsiveness and immune activation. During the control vs. persistent lymphocytosis stage, B cells display partial plasma cell–associated activation. Increased expression of *JCHAIN* (joining chain of multimeric immunoglobulins) is observed, suggesting enhanced immunoglobulin assembly. However, key transcriptional regulators of terminal plasma cell differentiation, including *PRDM1* (PR domain zinc finger protein 1; also known as B‐lymphocyte‐induced maturation protein 1, BLIMP1) and *XBP1* (X‐box binding protein 1), are not fully upregulated, indicating incomplete differentiation into antibody‐secreting plasma cells. In the persistent lymphocytosis vs. asymptomatic stage, attenuation of canonical BCR signaling and reduced antigen presentation are observed. This is reflected by decreased expression of *SYK* (spleen tyrosine kinase) and *CD79* (cluster of differentiation 79; Igα/Igβ components of the BCR complex), along with downregulation of MHC class II (major histocompatibility complex class II) genes, including *BoLA‐DRA* and *BoLA‐DRB3*. These changes are consistent with impaired immune surveillance and the emergence of immune evasion mechanisms during malignant progression. AC, Asymptomatic, CT, Control, PL, Persistent lymphocytosis. Arrows indicate relative upregulation (↑) or downregulation (↓) of gene expression compared with the preceding disease stage.

One of the most prominent findings in asymptomatic animals was the significant upregulation of proximal BCR signaling components, particularly *CD79B* and *SYK*, accompanied by increased MHC class II expression (*BoLA‐DRA* and *BoLA‐DRB3*). *CD79B* and *SYK* are essential mediators of antigen‐induced BCR activation and downstream signal propagation (Kurosaki et al. 2010; Reth 2014). Their coordinated upregulation suggests that BLV infection in its clinically silent phase is associated with increased B‐cell activation rather than early immune suppression (Figure 2).

This observation aligns with previous studies demonstrating that BLV preferentially infects and expands IgM^+^ CD5^+^ B cells, leading to persistent polyclonal B‐cell activation (Florins et al. 2007; Gillet et al. 2007). Similarly, transcriptomic analyses of BLV‐infected cattle with high proviral load have reported upregulation of immune activation pathways, including signaling cascades downstream of the BCR complex (Gonzalez‐Mendez et al. 2025; Petersen et al. 2025).

Beyond BLV, sustained BCR signaling has been shown to play a critical role in maintaining B‐cell survival during chronic viral infections and lymphoproliferative disorders. Thus, our findings suggest that asymptomatic BLV infection may represent a pre‐malignant state characterized by enhanced signaling competence and proliferative readiness.

### 4.2 Partial Plasma Cell–Associated Activation Without Terminal Differentiation

The significant upregulation of *JCHAIN*, in the absence of increased expression of *PRDM1* (BLIMP1) and *XBP1*, indicates partial activation of antibody‐associated machinery without full plasma cell differentiation. *JCHAIN* is required for multimeric IgM and IgA assembly and is typically expressed in antibody‐secreting cells (Brandtzaeg 2013). However, terminal plasma cell differentiation requires coordinated induction of *PRDM1* and *XBP1*, which orchestrate immunoglobulin secretion and unfolded protein response programs (Iwakoshi et al. 2003; Shapiro‐Shelef and Calame 2005).

The absence of significant *PRDM1* and *XBP1* upregulation in our dataset suggests that BLV infection promotes an activated B‐cell phenotype rather than terminal differentiation (Figure 2). Similar partial activation states have been described in chronic viral infections, where B cells display increased immunoglobulin‐related gene expression without acquiring full plasma cell identity (Moir and Fauci 2009). In BLV infection specifically, previous studies have reported hypergammaglobulinemia and altered humoral responses during persistent lymphocytosis without clear evidence of widespread plasma cell transformation (Marin‐Flamand et al. 2022; Trueblood et al. 1998).

Collectively, these findings indicate that BLV‐associated asymptomatic infection is marked by functional activation and immunoglobulin assembly readiness, but not by complete plasma cell maturation.

### 4.3 Attenuation of Canonical BCR Signaling in Persistence Lymphocytosis

Key observation in the transition from asymptomatic infection to PL was the significant downregulation of *CD79B* and *SYK* in malignant samples relative to asymptomatic animals. The observed transcriptional alterations in persistent lymphocytosis animals likely reflect stage‐dependent immune and functional reprogramming associated with advanced BLV infection rather than definitive malignant lymphoma transformation.

While many human B‐cell lymphomas depend on active BCR signaling for survival (Young and Staudt 2013), certain lymphoma subtypes exhibit altered or uncoupled BCR signaling pathways as part of oncogenic reprogramming (Davis et al. 2010). Experimental BLV models have similarly shown that viral integration and clonal expansion are accompanied by altered signaling and proliferation pathways (Gillet et al. 2007).

Reduced expression of proximal BCR components may reflect a shift from antigen‐driven activation to oncogene‐driven proliferation. In retrovirus‐associated malignancies, transformation is often accompanied by rewiring of signaling networks that reduce dependence on physiological receptor stimulation (Gillet et al. 2007). Thus, the attenuation of *CD79B* and *SYK* in PL samples may reflect stage‐dependent functional reprogramming associated with advanced BLV infection rather than simple immune exhaustion (Figure 2).

### 4.4 Germinal Center Program Is Not Prominently Engaged

Interestingly, germinal center–associated transcription factors (*BCL6, AICDA, RGS13, MEF2B*) did not show significant differential expression across comparisons. *BCL6* and *AICDA* are canonical regulators of germinal center reactions and somatic hypermutation (Victora and Nussenzweig 2012). In human lymphomas, particularly germinal center–derived DLBCL, these genes are frequently dysregulated (Basso and Dalla‐Favera 2015).

The absence of a strong germinal center signature in our dataset suggests that BLV‐associated lymphoma does not transcriptionally resemble a classical germinal center–derived malignancy. This observation is consistent with prior BLV research indicating that transformed cells originate primarily from peripheral CD5^+^ B cells rather than bona fide germinal center B cells (Florins et al. 2007). Our findings therefore support a model in which BLV‐driven oncogenesis arises from chronically activated peripheral B cells undergoing functional reprogramming rather than germinal center–dependent transformation.

### 4.5 Altered Antigen Presentation and Immune Evasion

The stage‐dependent modulation of *BoLA‐DRA* and *BoLA‐DRB3* expression further underscores dynamic immune remodeling during BLV infection (Figure 2). Upregulation of MHC class II genes in asymptomatic animals suggests enhanced antigen presentation and immune surveillance during early infection. MHC class II molecules are central to CD4^+^ T‐cell activation and antiviral immunity (Neefjes et al. 2011).

However, the observed reduction of these genes in lymphoma relative to asymptomatic animals suggests partial impairment of antigen presentation during malignant progression. Downregulation of MHC class II molecules is a well‐documented immune evasion mechanism in both human and animal lymphomas (Reichel et al. 2015; Steidl et al. 2011). Loss of antigen presentation capacity reduces T‐cell–mediated tumor recognition and facilitates immune escape.

The coordinated upregulation of *BoLA‐DRA* and *BoLA‐DRB3* in asymptomatic animals suggests enhanced MHC class II–mediated antigen presentation during early BLV infection. Notably, *BoLA‐DRB3* represents the major polymorphic and functionally relevant bovine MHC class II DR β‐chain locus associated with immune responsiveness and BLV susceptibility. Importantly, MHC class II molecules in cattle are expressed not only by B lymphocytes but also by activated T cells, monocytes/macrophages, and professional antigen‐presenting cells.

In BLV infection, previous studies have suggested that viral persistence is associated with immune modulation and impaired antiviral responses (Florins et al. 2007). Our data extend these findings by demonstrating that antigen presentation dynamics are not static but evolve across disease stages, potentially contributing to PL immune evasion.

The observed transcriptional alterations in persistent lymphocytosis animals likely reflect stage‐dependent immune and functional reprogramming associated with advanced BLV infection rather than definitive malignant lymphoma transformation.

Importantly, the present study investigated transcriptional alterations associated with asymptomatic infection and persistent lymphocytosis stages of BLV infection rather than overt lymphoma. None of the animals included in this study had histopathologically confirmed lymphosarcoma. Therefore, the identified transcriptional changes should be interpreted as stage‐dependent immune and functional remodeling associated with disease progression rather than definitive molecular signatures of progression to persistent lymphocytosis.

## 5 Conclusion

This study provides a focused transcriptomic dissection of B‐cell–associated immune programs across distinct stages of BLV infection and demonstrates a dynamic, stage‐dependent remodeling of B‐cell function. The asymptomatic phase is characterized by enhanced proximal BCR signaling, increased antigen presentation capacity, and partial activation of immunoglobulin assembly pathways, reflecting a state of heightened immune activation. In contrast, progression to persistent lymphocytosis is associated with attenuation of canonical BCR signaling components and reduced expression of antibody assembly and MHC class II genes, indicating functional reprogramming during progression to persistent lymphocytosis.

Importantly, the absence of significant modulation of germinal center transcription factors could suggest that BLV‐associated lymphomagenesis does not follow a classical germinal center–derived oncogenic pathway. Instead, the data support a model in which chronically activated peripheral B cells undergo progressive transcriptional reprogramming associated with viral persistence and lymphoproliferative disease progression. These findings contribute to a refined mechanistic understanding of BLV‐induced immune dysregulation and B‐cell oncogenesis.

## 6 Limitations

A limitation of the present study is that transcriptomic analyses were performed using peripheral blood lymphocytes (PBLs), which may not fully represent germinal center–specific transcriptional activity typically occurring within secondary lymphoid tissues such as lymph nodes and spleen. Therefore, the observed lack (or presence) of differential expression in germinal center–associated genes and transcription factors should be interpreted with caution, as it may reflect the sampling source rather than a true absence of germinal center–related biological processes during BLV infection.

Because transcriptomic analyses were performed on isolated peripheral blood leukocytes rather than directly stabilized whole blood, it is possible that leukocyte separation procedures and variations in cellular composition may have influenced the observed gene expression patterns. Although all samples were processed under standardized conditions to minimize technical variability, potential effects of sample handling on transcriptional profiles cannot be completely excluded.

Another limitation of the present study is the absence of animals with histopathologically confirmed lymphosarcoma. Therefore, the transcriptional alterations identified in persistent lymphocytosis animals should not be interpreted as definitive markers of progression to persistent lymphocytosis, but rather as molecular changes associated with advanced lymphoproliferative stages of BLV infection.

Because transcriptomic analyses were performed using total peripheral blood leukocytes rather than purified immune‐cell subsets, the relative contribution of B cells, T cells, monocytes/macrophages, and other antigen‐presenting populations to the observed MHC class II transcriptional changes could not be definitively resolved.

Future studies incorporating lymphoid tissue samples would be valuable to more accurately assess germinal center–specific immune responses.

## 7 Future Perspectives

Several avenues emerge from the present findings. First, functional validation of altered BCR signaling pathways through phosphoproteomic or single‐cell analyses would clarify whether transcriptional attenuation in lymphoma reflects signaling exhaustion, oncogenic rewiring, or clonal selection of signaling‐independent PBL. Second, integration of proviral load quantification with B‐cell transcriptional signatures may further elucidate how viral burden shapes immune remodeling across disease stages. Third, longitudinal studies tracking asymptomatic animals over time could determine whether early BCR hyperactivation predicts subsequent progression to persistent lymphocytosis.

Additionally, characterization of tumor microenvironment interactions, particularly CD4^+^ T‐cell engagement and antigen presentation competence, may reveal mechanisms of immune escape during lymphoma progression. Finally, identification of stage‐specific transcriptional biomarkers may provide novel diagnostic tools for early detection of malignant transition and inform the development of targeted therapeutic strategies aimed at restoring physiological B‐cell signaling and immune surveillance in BLV‐infected cattle.

## Author Contributions

All authors contributed to the conception of the study, and Mohammad Mehdi Akbarin and Hugo Ramírez Álvarez designed it. Mohammad Mehdi Akbarin, Zahra Farjami, Gabriel Eduardo Acevedo‐Jiménez, Cecilia Rodríguez Murillo, Víctor David González‐Fernández, Lucero de María Ávila‐De la Vega, and Hugo Ramírez Álvarez performed data collection and analysis. Mohammad Mehdi Akbarin and Zahra Farjami wrote the initial draft of the manuscript, and all authors provided comments on subsequent versions. Mohammad Mehdi Akbarin and Hugo Ramírez Álvarez edited the final draft. All authors read and approved the final manuscript.

## Funding

The authors have nothing to report.

## Ethics Statement

Institutional Review Board Statement: The animal study protocol was approved by the Institutional Review Board of Internal Committee on Animal Use and Experimentation (CICUAE‐FESC) of the National Autonomous University of Mexico (code CICUAE‐FESC. NC 23_35/05‐2023) on 05 August 2023.

## Consent

The authors have nothing to report.

## Conflicts of Interest

The authors declare no conflicts of interest.

## Supporting information


**File S1:** Differential gene expression profiles identified among BLV‐infected cattle clinical groups. Comprehensive list of differentially expressed genes (DEGs) obtained from transcriptomic analysis comparing Control versus Asymptomatic (AL), Control versus Persistent Lymphocytosis (PL), and PL versus Asymptomatic cattle. The file includes gene symbols, log fold‐change (logFC) values, and adjusted *p*‐values (FDR‐corrected) for each comparison. Positive logFC values indicate upregulation in the second group of each comparison, whereas negative values indicate downregulation. These data provide the complete set of significant transcriptional alterations associated with bovine leukemia virus (BLV) infection and disease progression, supporting the analyses presented in the main manuscript.

## Data Availability

No applicable (this manuscript does not report data generation or analysis).
